# Deriving a Chronic Guideline Value for Nickel in Tropical and Temperate Marine Waters

**DOI:** 10.1002/etc.4880

**Published:** 2020-11-10

**Authors:** Francesca Gissi, Zhen Wang, Graeme E. Batley, Kenneth M.Y. Leung, Christian E. Schlekat, Emily R. Garman, Jenny L. Stauber

**Affiliations:** ^1^ CSIRO Oceans and Atmosphere, Lucas Heights, New South Wales Australia; ^2^ Institute of Marine Sciences and Guangdong Provincial Key Laboratory of Marine Biotechnology, Shantou University Shantou China; ^3^ CSIRO Land and Water, Lucas Heights, New South Wales Australia; ^4^ State Key Laboratory of Marine Pollution and Department of Chemistry, City University of Hong Kong, Tat Chee Avenue, Kowloon Hong Kong China; ^5^ NiPERA Durham North Caroline USA

**Keywords:** Water quality criteria, Saltwater, Aquatic toxicity, Species sensitivity distribution, Hazardous concentration, Metals

## Abstract

The absence of chronic toxicity data for tropical marine waters has limited our ability to derive appropriate water quality guideline values for metals in tropical regions. To aid environmental management, temperate data are usually extrapolated to other climatic (e.g., tropical) regions. However, differences in climate, water chemistry, and endemic biota between temperate and tropical systems make such extrapolations uncertain. Chronic nickel (Ni) toxicity data were compiled for temperate (24 species) and tropical (16 species) marine biota and their sensitivities to Ni compared. Concentrations to cause a 10% effect for temperate biota ranged from 2.9 to 20 300 µg Ni/L, with sea urchin larval development being the most sensitive endpoint. Values for tropical data ranged from 5.5 to 3700 µg Ni/L, with copepod early–life stage development being the most sensitive test. There was little difference in temperate and tropical marine sensitivities to Ni, with 5% hazardous concentrations (95% confidence interval) of 4.4 (1.8–17), 9.6 (1.7–26), and 5.8 (2.8–15) µg Ni/L for temperate, tropical, and combined temperate and tropical species, respectively. To ensure greater taxonomic coverage and based on guidance provided in Australia and New Zealand, it is recommended that the combined data set be used as the basis to generate a jurisdiction‐specific water quality guideline of 6 µg Ni/L for 95% species protection applicable to both temperate and tropical marine environments. *Environ Toxicol Chem* 2020;39:2540–2551. © 2020 The Authors. *Environmental Toxicology and Chemistry* published by Wiley Periodicals LLC on behalf of SETAC.

## INTRODUCTION

Nickel (Ni) exposure in marine waters occurs from many anthropogenic and natural sources, of which mining is a particular concern (Hédouin et al. 2016). Nickel is predominantly mined from 2 main ore types, magmatic sulfides, typical of colder climates (e.g., Russia and Canada), and lateritic ores, which are formed from the extensive chemical and physical weathering of ultramafic rock, common in tropical regions (Mudd 2010). Recent estimates show that 60% of the world's Ni reserves are contained in laterite deposits (US Geological Survey 2019). In 2018, 48% of the world's Ni production came from the tropical Asia‐Pacific region, including Indonesia, New Caledonia, and the Philippines (US Geological Survey 2019).

Given the increase in production of Ni from tropical regions, there is a concern that tropical environments may be at an increased risk of exposure from Ni mining activities. Uncertainty may be particularly high among developing nations, where risk‐assessment procedures and regulatory frameworks are less developed than in temperate regions (Gissi et al. 2016). Tropical environments are unique and highly biodiverse compared to temperate regions (Howe et al. 2012). Mangroves, seagrasses, and coral reefs provide habitats which support the biodiversity of other marine life including primary producers, zooplankton, larger crustaceans, mollusks, echinoderms, and fish (Hoeksema 2007). Tropical systems differ from temperate systems because of their warmer temperatures, lower dissolved oxygen, high irradiance, high rainfall, strong rainfall seasonality, and more frequent pulse events such as cyclones/typhoons. Periods of high rainfall increase runoff from catchments that can potentially cause a higher influx of contaminants, nutrients, and sediments into the coastal marine environment (Hunter and Walton 2008).

Concentrations of Ni in unimpacted surface marine coastal waters are typically <0.2 µg/L (Apte et al. 2018). For unimpacted European waters, Heijerick and Van Sprang (2008) reported 90th percentile monitoring values of 3.3 and 0.3 µg Ni/L for estuarine/coastal and open ocean waters, respectively. In some regions, such as New Caledonia, Ni concentrations in soils and aquatic systems are naturally enriched; but mining of lateritic Ni ores can result in additional input of metals into the surrounding coastal system (Hedouin et al. 2009). Dissolved Ni concentrations in seawater in New Caledonia have been reported in the range <0.1 to 11 µg Ni/L (Moreton et al. 2009).

The question arises as to whether water quality guideline values derived for temperate regions will be applicable (or protective) to tropical marine ecosystems. Several studies have investigated the differences in sensitivities of saltwater species to chemical contaminants across different climatic regions. Chapman et al. (2006) showed that tropical marine invertebrates were no more or less sensitive to 4 metals (copper, cadmium, zinc, and lead) than their temperate counterparts based on differences in species sensitivity distributions (SSDs) of acute toxicity data. Similarly, Wang et al. (2014) found only small differences in the acute toxicity of chemicals between tropical and temperate marine biota. For Ni, they showed that hazardous concentrations for 10% of species (HC10) were 658 (95% CI 557–767) µg Ni/L for temperate species (*n* = 49) and 1560 (95% CI 366–3060) µg Ni/L for tropical species (*n* = 8). Although this suggests that tropical species may be less acutely sensitive to Ni than temperate species, there is considerable uncertainty (overlap of the 95% CI). There were also significant differences in the amount of toxicity data available for temperate and tropical regions; 49 temperate species versus 8 tropical species (Wang et al. 2014).

A recent study by Peters et al. (2019) compared the chronic toxicity of Ni for temperate (*n* = 19) and tropical (*n* = 16) freshwater species. They found little difference in the sensitivities of temperate and tropical freshwater species to Ni and recommended combining temperate and tropical data sets to include the most diverse range of taxa possible to ensure the protection of sensitive species across both regions. Similar comparisons with chronic marine Ni data have not been published, and only recently has the toxicity of Ni to key unique tropical taxa such as corals been reported (Gissi et al. 2017, 2018; Wang et al. 2020).

The scarcity of chronic toxicity data for marine waters (Gissi et al. 2016) has limited our ability to conduct robust risk assessments or to derive appropriate guideline values for tropical regions. More usually, temperate data are simply applied to meet environmental management needs. Differences in physicochemical parameters, such as temperature, rainfall, and irradiance, between temperate and tropical systems make such extrapolations highly uncertain. Therefore, the aim of the present study was to compile existing and newly generated chronic Ni toxicity data for temperate and tropical marine organisms, to determine if there were significant differences in sensitivity to Ni between these 2 geographical groups and to derive a guideline value for Ni in marine waters for either or both climatic regions.

## METHODS

### Definition of temperate and tropical species

For the present study, temperate biota were defined as species isolated from temperate regions and/or having a natural geographical distribution outside of the Tropics of Cancer and Capricorn, and toxicity tests were conducted at temperatures <25 °C. Tropical biota were defined as species isolated from tropical regions and/or having a natural geographical distribution between the Tropics of Cancer and Capricorn, and toxicity tests were conducted at temperatures ≥25 °C. Data were considered marine and included in the compilation if the species was found in, and the test was conducted in, salinities ≥25‰ (Warne et al. 2018).

### Compilation and quality check of data

Existing Ni toxicity data for tropical and temperate species were compiled from the literature (up to 2018) following searches in databases including Web of Science, Scopus, and Google Scholar. The data preference was for chronic 10% effect concentration (EC10) and 10% inhibition concentration (IC10) values rather than no‐observed‐effect concentration (NOEC) values. In some instances, only EC50 and IC50 values (the concentration that causes a 50% effect or inhibition relative to the control) and lowest‐observed‐effect concentration (LOEC) values were available. Chronic toxicity is defined as an adverse effect that occurs after exposure for a substantial portion of the organism's life span (usually > 10%) or an adverse sublethal effect on a sensitive early life stage (e.g., fertilization over 5 h; Batley et al. 2018; Warne et al. 2018). The species and life stage of the test organisms, exposure duration, and test endpoint (e.g., survival, growth, fertilization) were recorded. In addition, key water quality parameters such as test temperature, pH, dissolved organic carbon (DOC) and salinity were compiled for each test.

Prior to use in the SSDs, all data were quality‐checked using a data quality checklist (Australian and New Zealand Governments 2018; Warne et al. 2018). We followed guidance provided by Warne et al. (2018) and included data that scored ≥50% in the SSDs. To achieve a score ≥50%, criteria to be met in the checklist of Warne et al. (2018) included, but were not limited to, use of appropriate controls, replication of controls and contaminant concentrations, inclusion of reference toxicant, stated test acceptability criteria, description of test organism (e.g., life stage, length, mass, age), measurement of contaminant concentrations, measurement of water quality parameters, and use of appropriate statistical method to determine toxicity.

### SSDs

Data were fitted to SSDs using the Burrlioz 2.0 software (Australian and New Zealand Governments 2018). If insufficient chronic EC, IC, and lethal concentration (LC; 10–20) and NOEC data were available, chronic LOEC and EC/IC/LC50 data were converted to chronic NOEC values by dividing by factors of 2.5 and 5, respectively, according to the method of Warne et al. (2018). These conversions were required; otherwise, EC50 and LOEC data would need to be excluded from the chronic SSDs. Where there was more than one value reported for the same species and endpoint, the geometric mean was calculated and included in the SSD. Where there were multiple values for the same species using different endpoints, the most sensitive endpoint (i.e., the lowest toxicity value) was used. Chronic toxicity data sets with 8 or more data were fitted to a Burr type III distribution, and the HC1, HC5, HC10, and HC20 values determined. In Australia and New Zealand, the HC5 (i.e., 95% protective concentration [PC95]) value applies to slightly to moderately disturbed systems (i.e., most systems), whereas the HC1 (i.e., PC99) is applied to systems of high ecological value (Australian and New Zealand Governments 2018).

### Comparison of species sensitivity

Pairwise temperate and tropical SSD comparisons were conducted by a combination of statistical tests and visual inspection (Wang et al. 2014). Analysis of covariance (ANCOVA; SPSS) was conducted to compare the slopes of the 2 SSDs based on a log‐normal distribution. A visual comparison of either congruence or discrepancy of the temperate and tropical distributions was also used by plotting the cumulative distribution of the Ni toxicity data in Excel (Supplemental Data, Figure S1). If the 95% CIs of the 2 HC5 values (e.g., temperate vs tropical SSDs) did not overlap, then the 2 SSDs were considered significantly different (Wang et al. 2014). The toxicity data for each phylum from temperate and tropical regions were displayed in box plots, created in the statistical package NCSS (2007, Ver 07.1.21). Toxicity data for crustacea from temperate and tropical regions were compared using analysis of variance (ANOVA) in the statistical software package NCSS.

The formula of Litchfield and Wilcoxon (1949) was also used to compare the HC5 values derived from each SSD (temperate, tropical, and combined), to determine if there were any significant differences in the values calculated from the different data sets. Ratios between the HC5 values from temperate, tropical, and combined SSDs were calculated to compare the HC5 values calculated from each SSD. The upper limit of the 95% CI of the HC5 value calculated from each SSD (temperate, tropical, and combined) was also used to calculate the *F* ratio. If the ratio of the HC5 values was less than the *F* ratio, there was no significant difference in the HC5 values.

## RESULTS AND DISCUSSION

### Temperate and tropical marine toxicity data

Of the chronic toxicity data that scored ≥50% and were deemed applicable for water quality guideline development, there were 24 temperate species and 16 tropical species, giving a combined data set of 40 species representing 14 taxonomic groups (based on phyla; Warne et al. 2018) including diatoms, green algae, a dinoflagellate, a brown‐golden alga, cyanobacteria, copepods, a brown macroalga, a red macroalga, polychaetes, crustaceans, bivalve mollusks, gastropod mollusks, echinoderms, cnidarians (corals, sea anemone), and fish (Table 1). The tropical data set did not have data for macroalgae and bivalves, but it had 3 values for corals which mainly occur in tropical regions. In general, the species compositions between the 2 regions were comparable, with 6 out of 9 (67%) taxonomic groups in common.

**Table 1:** Taxonomic groups and numbers of species represented in the temperate and tropical datasets that were used to generate species sensitivity distributions

	No. species	
Taxonomic group—phylum	Temperate	Tropical
Cyanobacteria	0	1
Bacillariophyte	1	1
Haptophyte	0	1
Chlorophyte	1	0
Dinoflagellate	0	1
Rhodophyte	1	0
Ochrophyte	1	0
Crustacean	6	4
Echinoderm	6	1
Mollusk (gastropod)	1	2
Mollusk (bivalve)	4	0
Cnidarian	0	3
Annelid	1	1
Chordate	2	1
Total no. species	24	16

### Temperate species sensitivity to Ni

**Figure 1:**
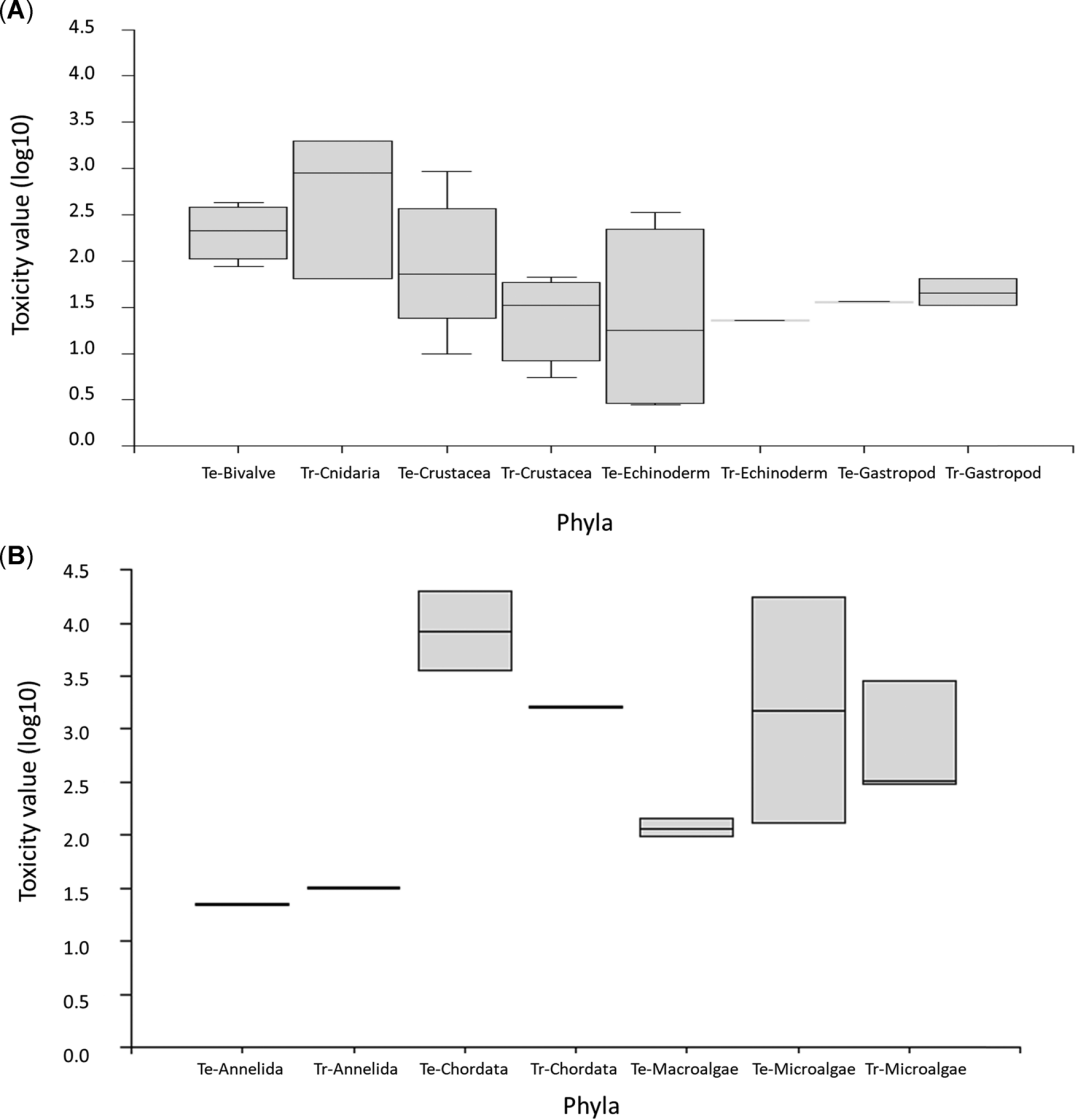
Box plots for invertebrates (**A**) and annelids, chordates, and macro‐/microalgae (**B**), representing the toxicity of nickel to groups (based loosely on phylum) of organisms from temperate and tropical regions. Note toxicity values, presented in Tables 2 and 3, were log‐transformed for graphical representation; when *n* > 3, box = median and interquartile range, whiskers = maximum and minimum values; when *n* ≤ 3, box = mean and maximum and minimum values—refer to Table 1. Te = temperate; Tr = tropical.

**Figure 2:**
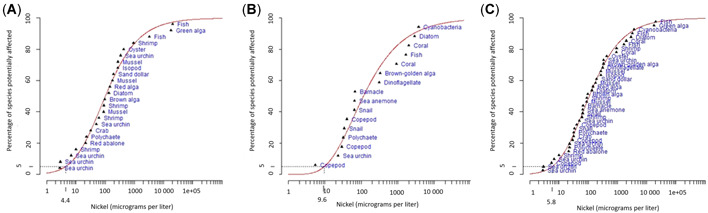
Species sensitivity distributions for (**A**) temperate marine species, (**B**) tropical marine species, and (**C**) combined temperate and tropical species. The dotted line indicates the 5% hazardous concentration value.

The sensitivity of temperate marine species to Ni varied widely across different taxonomic groups, with threshold and no effects (EC10/NOEC values) observed between 2.9 and 20 300 µg Ni/L (Table 2). The most sensitive species were echinoderms (sea urchins; EC10/NOEC values 2.9–500 µg Ni/L), polychaetes (reproduction inhibited by 10% at 23 µg Ni/L), gastropods (EC10/NOEC values for growth inhibition 21–36 µg Ni/L), and mysid shrimps (neonate survival [48 h] inhibited by 10% at 45 µg Ni/L; Table 2 and Figure 1A and B; Supplemental Data, Table S1). For sea urchins, the most sensitive endpoint was larval development, with EC10 values between 2.9 and 335 µg Ni/L (Hwang et al. 2012; DeForest and Schlekat 2013; Blewett et al. 2016). Fertilization tended to be less sensitive, with NOEC and EC50 values of 500 and 217 µg Ni/L, respectively for the urchin *Paracentrotus lividus* (Novelli et al. 2003; Pagano 2007). These studies used different species and different test conditions, so caution must be taken when making comparisons. Fish and green algae were the least sensitive taxa to Ni (Figure 1B), with 10% effects on fish larval survival and algal growth above 17 000 µg Ni/L. All temperate toxicity data, including values used in the SSDs are presented in Table 2. The complete data set, including details on test medium and physicochemical parameters (temperature, salinity, pH), is given in Supplemental Data, Table S1. Of the entire data set, 24 values (i.e., species) representing 10 taxonomic groups were selected for input into the SSDs (Figure 2A).

**Table 2:** Chronic nickel toxicity data for temperate marine species used in the species sensitivity distribution

Taxonomic group—phylum	Common name used in SSD	Species	Life stage	Duration	Toxicity measure	Reported toxicity value (µg/L)	Toxicity value used in SSD (µg/L)	Reference
Bacillariophyte	Diatom	*Skeletonema costatum*	—	96 h	EC10 (growth)	142	132^b^	Deforest and Schlekat (2013)
					EC10 (growth)	89		
					EC10 (growth)	383		
					EC10 (growth)	190		
					EC10 (growth)	43.5		
Chlorophyte	Green alga	*Dunaliella tertiolecta*	—	96 h	EC10 (growth)	17 890	17 900	Deforest and Schlekat (2013)
Rhodophyte	Red alga	*Champia parvula*	Adult	10 d	EC10 (reproduction)	144	144	Deforest and Schlekat (2013)
Ochrophyte	Brown macroalga	*Macrocystis pyrifera*	Zoospores	10 d	EC10 (germination)	494	97	Golder Associates (2007)
					EC10 (reproduction)	96.7		
Crustacean	Shrimp	*Mysidopsis intii*	Neonate	48 h	NOEC (survival)	10	10	Hunt et al. (2002)
					EC10 (survival)	45.2^a^		
Crustacean	Shrimp	*Mysidopsis bahia*	Larvae	36 d	EC50 (reproduction)	93	61	Gentile et al. (1982)
					NOEC (reproduction)	61		
				20 d	MATC (reproduction)	93		
Crustacean	Shrimp	*Artemia salina*	Eggs	48 h	EC50 (hatching rate)	4660	932^c^	Kissa et al. (1984)
				48 h	LOEC (hatching rate)	2770		
Crustacean	Shrimp	*Litopenaeus vannamei*	Postlarval	30 d	EC50 (mortality)	446	89^c^	Leonard et al. (2011)
Crustacean	Isopod	*Excirolana armata*	Postlarval	15 d	EC50 (survival)	1350	270^c^	Leonard et al. (2011)
Crustacean	Crab	*Portunus pelagicus*	Larvae	42 d	Mean of NOEC and LOEC (reduced size, molt inhibition)	32	32	Mortimer and Miller (1994)
Echinoderm	Sea urchin	*Diadema antillarum*	Larvae	40 h	EC50 (larval development)	15	2.9	Bielmyer et al. (2005)
					EC10 (larval development)	2.9^a^		
Echinoderm	Sea urchin	*Paracentrotus lividus*	Embryo	72 h	EC50 (fertilization)	217	50	Pagano (2007)
					NOEC (fertilization)	500		Novelli et al. (2003)
					NOEC (larval development)	50		
					EC50 (larval development)	320		
Echinoderm	Sea urchin	*Evechinus chloroticus*	Embryo	96 h	EC50 (larval development)	14	2.8^c^	Blewett et al. (2016)
Echinoderm	Sea urchin	*Hemicentrotus pulcherrimus*	Embryo	64 h	NOEC (larval development)	<10	6.8^c^	Hwang et al. (2012)
					LOEC (larval development)	25		
					EC50 (larval development)	34.2		
Echinoderm	Sea urchin	*Strongylocentrotus purpuratus*	Embryo	48 h	EC10 (larval development)	335	335	Deforest and Schlekat (2013)
Echinoderm	Sand dollar	*Dendraster excentricus*	Embryo	48 h	EC10 (larval development)	191	191	Deforest and Schlekat (2013)
Mollusk (Gastropod)	Abalone	*Haliotis rufescens*	Embryo	14 d	NOEC (shell growth)	21.5	21.5	Hunt et al. (2002)
					EC10 (shell growth)	36.4^a^		
Mollusk (Bivalve)	Oyster	*Crassostrea gigas*	Embryo	96 h	EC10 (reproduction)	431	431	Deforest and Schlekat (2013)
Mollusk (Bivalve)	Mussel	*Mytilus edulis*	Embryo	96 h	EC50 (development)	891	178^c^	Martin et al. (1981)
Mollusk (Bivalve)	Mussel	*Mytilus trossolis*	Embryo	48 h	EC20 (survival)	88	88	Nadella et al. (2009)
Mollusk (Bivalve)	Mussel	*Mytilus galloprovincialis*	Embryo	48 h	EC10 (survival)	259	270^b^	Deforest and Schlekat (2013)
					EC10 (survival)	228		
					EC10 (survival)	256		
					EC10 (survival)	350		
Annelid	Polychaete	*Neanthes arenaceodentata*	Adult	90 d	EC10 (reproduction)	22.5	22.5	Deforest and Schlekat (2013)
Chordate	Fish	*Atherinops affinis*	Embryo	40 d	NOEC (larval survival)	3240	3240	Hunt et al. (2002)
					EC10 (larval survival)	3600		
Chordate	Fish	*Cyprinidon variegatus*	Juvenile	28 d	EC10 (growth)	20 300	20 300	Golder Associates (2007)

^a^ The EC10 value is from DeForest and Schlekat (2013) using data supplied by authors.

^b^ Geometric mean.

^c^ Chronic EC50 converted to NOEC value by dividing by 5 (Warne et al. 2018).

EC10 = 10% effect concentration; LOEC = lowest‐observed‐effect concentration; MATC = maximum acceptable toxicant concentration; NOEC = no‐observed‐effect concentration; SSD = species sensitivity distribution.

### Tropical species sensitivity to Ni

Concentrations of Ni that caused a 50% effect to tropical species ranged from 6.6 to 22 500 µg Ni/L. Similar to the temperate data set, sea urchins and crustaceans were the most sensitive taxa to Ni, whereas algae were the least sensitive (Figure 1A and B). The most sensitive tropical marine species was the copepod *Acartia sinjiensis*, with an EC10 for development of 5.5 (5.0–6.0) µg Ni/L (Gissi et al. 2018). The EC10/NOEC values for other crustacea, including another species of copepod and a barnacle, ranged from 29 to 99.8 µg Ni/L. Endpoints included mortality, development/metamorphosis, and intrinsic rate of population increase (Gissi et al. 2018; Wang et al. 2020). Only one species of tropical sea urchin has been reported in the literature, with a NOEC for normal larval development of 23 µg Ni/L (Rosen et al. 2015). Gastropods (snails) were also relatively sensitive to Ni, with EC10 values for growth rate inhibition at concentrations of 33 to 64 µg Ni/L (Gissi et al. 2018; Wang et al. 2020). Cyanobacteria, diatoms, dinoflagellates, and a brown alga were some of the least sensitive species (Figure 1B), with EC10 values between 330 and 3700 µg Ni/L (Alquezar and Anastasi 2013; Gissi 2018). Coral was one of the least sensitive species, with an EC10 for fertilization success of 2000 µg Ni/L (Gissi et al. 2017). All tropical toxicity data, including values used in the SSDs, are presented in Table 3. The complete data set, including details on test medium and physicochemical parameters (temperature, salinity, pH) is presented in Supplemental Data, Table S2. Of the entire data set, 16 values (i.e., species) were compiled for input into the SSDs representing 10 taxonomic groups (Figure 2B).

**Table 3:** Chronic nickel toxicity data for tropical marine species used in the species sensitivity distribution

Taxonomic group—phylum	Common name used in SSD	Species	Life stage	Duration	Toxicity measure	Reported toxicity value (µg/L)	Toxicity value used in SSD (µg/L)	Reference
Cyanobacteria	Cyanobacteria	*Cyanobium* sp.	6 × 10^3^ cells/mL	72 h	EC10 (growth rate)	3700	3700	Alquezar and Anastasi (2013)
					EC50 (growth rate)	22 500		
Bacillariophytea	Diatom	*Ceratoneis closterium (G2)* a	5–6 d old, 1–3 × 10^3^ cells/mL	72 h	NOEC (growth rate)	3970	2870^d^	Gissi (2018)
					EC10 (growth rate)	3250		
		*Ceratoneis closterium (F2)* b			NOEC (growth rate)	1610		
					EC10 (growth rate)	2539		
Haptophyte	Brown‐golden alga	*Tisochrysis lutea*	5–6 d old, 1–3 × 10^3^ cells/mL	72 h	NOEC (growth rate)	250	330	Gissi (2018)
					EC10 (growth rate)	330		
Miozoan	Dinoflagellate	*Symbiodinium* sp. Freud. Clade C.	6–7 d old, 1–3 × 10^3^ cells/mL	72 h	NOEC (growth rate)	310	310	Gissi (2018)
Crustacean	Barnacle	*Amphibalanus amphitrite*	Nauplii (<2 h old)	96 h	EC20 (metamorphosis)	97	67	Gissi et al. (2018)
					EC10 (metamorphosis)	67		
Crustacean	Copepod	*Acartia sinjiensis*	Egg	80 h	EC20 (development)	6.6	5.5	Gissi et al. (2018)
					EC10 (development)	5.5		
Crustacean	Copepod	*Acartia pacifica*	Adult females	10 d	LOEC (egg production)	100	40^f^	Mohammed et al. (2010)
Crustacean	Copepod	*Tigriopus japonicus*	Nauplii (<24 h old)	20–30 d	LC10 (mortality)	484	29	Wang et al. (2020)
					NOEC (mortality)	99.8		
					LOEC (mortality)	200		
					LOEC (mortality)	99.8		
					NOEC (mortality)	50.3		
					LC10 (mortality)	43.9		
					EC10 (intrinsic rate of increase)^c^	29.1		
					EC20 (intrinsic rate of increase)	66.5		
					EC50 (intrinsic rate of increase)	277		
					NOEC (intrinsic rate of increase)	50.3		
					LOEC (intrinsic rate of increase)	99.8		
Mollusk (gastropod)	Snail	*Nassarius dorsatus*	Larvae (2 d old)	96 h	EC20 (growth rate)	143	64	Gissi et al. (2018)
					EC10 (growth rate)	64		
Mollusk (gastropod)	Snail	*Monodonta labio*	Juvenile (<10 d old)	30 d	LC10 (mortality)	57	34	Wang et al. (2020)
					EC10 (growth rate)	33.6		
					EC20 (growth rate)	58.5		
					EC50 (growth rate)	151		
					NOEC (growth rate)	21.7		
					LOEC (growth rate)	53.9		
					EC10 (shell length increment)	93.5		
					EC20 (shell length increment)	145		
					EC50 (shell length increment)	308		
					NOEC (shell length increment)	53.9		
					LOEC (shell length increment)	107		
Cnidarian	Coral	*Acropora digitifera*	Gametes	5 h	NOEC (fertilization)	940	2000	Gissi et al. (2017)
					EC10 (fertilization)	2000		
					EC5 (fertilization)	1680		
Cnidarian	Coral	*Platygyra daedalea*	Gametes	5 h	NOEC (fertilization)	920	920	Gissi et al. (2017)
Cnidarian	Coral	*Platygyra daedalea*	Gametes	5 h	EC50 (fertilization)	1420		Reichelt‐Brushett and Hudspith (2016)
Cnidarian	Sea anemone	*Exaiptasia pulchella*	Lacerate tentacle	14 d	EC10 (development)	260	65	Howe et al. (2014)
			Adult	28 d	EC10 (reproduction—total no. offspring)	260		
					EC50 (reproduction—total no. offspring)	400		
					LOEC (reproduction—total no. offspring)	510		
					EC10 (reproduction—total no. juveniles)	65		
					EC50 (reproduction—total no. juveniles)	370		
					LOEC (reproduction—total no. juveniles)	510		
Echinoderm	Sea urchin	*Diadema savignyi*	Gametes	48 h	EC50 (fertilization and development)	117	23^d^	Rosen et al. (2015)
					LOEC (fertilization and development)	36.5		
					NOEC (fertilization and development)	23.5		
					EC50 (fertilization and development)	71.6		
					LOEC (fertilization and development)	36.5		
					NOEC (fertilization and development)	22.5		
Annelid	Polychaete	*Hydroides elegans*	Gametes	1 h	EC50 (sperm viability/fertilization)	773	32^e^	Gopalakrishnan et al. (2008)
					EC50 (egg viability/fertilization)	1178		
					EC50 (embryo development)	2263		
			Adults	20 h	EC50 (larval release)	410		
			Larvae	96 h	EC50 (larval settlement)	160		
Chordate	Fish	*Oryzias melastigma*	Juvenile	21 d	LC10 (mortality)	1660	1660	Wang et al. (2020)
					LC20 (mortality)	2310		
					LC50 (mortality)	4060		

^a^ Previously known as *Nitzschia closterium*, grown in G2 media (Loeblich and Smith 1968).

^b^ Previously known as *Nitzschia closterium*, grown in F2 media (Guillard and Ryther 1962).

^c^ Intrinsic rate of increase = population growth = number of births – number of deaths.

^d^ Geometric mean.

^e^ Chronic EC50 converted to NOEC value by dividing by 5 (Warne et al. 2018).

^f^ Chronic LOEC converted to NOEC value by dividing by 2.5 (Warne et al. 2018).

EC10 = 10% effect concentration; LC10 = 10% lethal concentration; LOEC = lowest‐observed‐effect concentration; NOEC = no‐observed‐effect concentration; SSD = species sensitivity distribution.

### Comparison of species sensitivity

There was little difference in the sensitivities of temperate and tropical species to Ni (Figure 1A and B). Comparisons are limited to phyla where toxicity data were available for more than one species for each group. For example, the ranges of toxicity values for crustacea (6 temperate species, 4 tropical species) overlap in the box plots (Figure 1A), and there was no significant difference in the range of toxicity values (ANOVA, *p* = 0.22). Temperate and tropical microalgae also have a similar range of sensitivities (Figure 1B). Because of the uneven representation of taxonomic groups and species across the temperate and tropical data sets, it is difficult to make direct comparisons of species sensitivities to Ni across different climatic regions as was done for freshwaters by Peters et al. (2019). There were more chronic Ni toxicity data available for temperate freshwater species (31 species; Peters et al. 2019) compared to 24 temperate marine species. The number of tropical data was similar, with 13 freshwater tropical species used by Peters et al. (2019) and 16 tropical marine species in the present study.

The log‐normal and Burr type III distributions were fitted to the SSDs, and the resulting HC*x* values calculated from these distributions were compared (Supplemental Data, Table S3). There was no significant difference in the HC values calculated from either distribution. This is further supported by the study of Wang et al. (2014), which showed little difference in a range of different distributions applied to acute marine toxicity data for several different toxicants. Requirements around SSDs and the preference for model selection are jurisdiction‐dependent. The present study has followed guidance described in Warne et al. (2018) and Batley et al. (2018), and the objective was to develop a guideline value for the region; therefore, the Burr type III distribution was ultimately selected to derive the final HC*x* values.

Tropical SSDs (Figure 2B) were found to be significantly different from the temperate and the combined SSDs, as indicated by significantly different slope parameters (ANCOVA *p* < 0.05; Supplemental Data, Table S4) and the crossover of the distributions (Supplemental Data, Figure S1; Wang et al. 2014). The temperate SSD was also found to be different from the combined SSD (ANCOVA *p* < 0.05 for different slopes; Supplemental Data, Table S4 and Figure S1). However, tropical and temperate species shared a similar sensitivity to Ni (Figure 1). In addition, there was no significant difference in the HC5 values derived from each SSD, temperate, tropical, or combined (Figure 2A–C) as reflected by the overlapping 95% CIs (Table 4). This was supported by calculations using the Litchfield‐Wilcoxon formula, which showed that there was no statistically significant difference between the HC5 values obtained from all SSDs. For all 3 comparisons, the ratios of the HC5 values were less than the *F* ratio (temperate vs tropical, 2.13 < 3.3; temperate vs combined, 1.29 < 3.2; tropical vs combined, 1.66 < 2.3).

**Table 4:** Toxicity values for nickel for marine ecosystems

	Toxicity value, µg Ni/L (95% confidence interval)		
Protection level (HC; %)	Temperate	Tropical	Temperate + tropical
1%	1.2 (0.14–7.4)	4.6 (0.03–15)	1.8 (0.43–7.2)
5%	4.4 (1.8–17)	9.6 (1.7–26)	5.8 (2.8–15)
10%	8.7 (3.9–27)	15 (7.1–41)	11 (5.6–25)
20%	20 (8.1–53)	28 (13–89)	23 (12–47)

HC = hazardous concentration.

A range of factors other than geographical distribution and number of taxa limits our ability to compare the sensitivities of tropical and temperate species to Ni. Differences in temperature between temperate and tropical tests may affect both toxicokinetics and toxicodynamic processes (Zhou et al. 2014), although species are likely adapted to the temperature conditions of their environment. Temperate species were typically tested in the range of 15 to 24 °C, whereas tropical species were tested between 25 and 30 °C. It is important to note that species were tested at temperatures to which they had been acclimated. For this reason, it is unlikely that temperature plays a significant role in Ni toxicity, and the response of organisms to Ni exposure at temperatures outside of their normal range may not be as we expect. For example, Pereira et al. (2017) showed that Ni toxicity to the freshwater flea *Daphnia magna* increased as temperature decreased.

Nickel is known to be an essential nutrient for microorganisms and terrestrial plants, but essentiality in aquatic animals has not been confirmed (Muyssen et al. 2004; Moreton et al. 2009). In freshwater temperate biota, Ni is thought to be a respiratory toxicant (acute exposures to fish and some invertebrates), an ionoregulatory toxicant (invertebrates), and a promoter of oxidative stress (Brix et al. 2017). Mechanisms of Ni toxicity, particularly chronic effects, in the marine environment, however, are not well understood, with only limited studies on temperate killifish, copepods, mussels, and the green shore crab, looking at acute effects at high Ni concentrations (Blewett and Leonard 2017). Only 2 mechanistic studies have investigated tropical species, and both only examined acute toxicity to freshwater species. Nath and Kumar (1989) found effects of Ni on the gills of the tropical perch at very high Ni concentrations (13 mg Ni/L) over a 96‐h exposure at 24 °C, whereas Palmero et al. (2015) found that Ni at 2.5 mg/L affected antioxidant defenses in the freshwater fish *Prochilodus lineatus* (but at 20 °C). Consequently, there are insufficient data to determine whether Ni has a different mode of action to tropical species at higher temperatures than to temperate marine species.

Water quality parameters, such as salinity and DOC, can also influence the toxicity of Ni to marine biota, although the effects of DOC may be small (Blewett et al. 2018). Blewett et al. (2016, 2018) showed that Ni toxicity to the urchin *Evechinus chloroticus* and the mussel *Mytilus edulis* was influenced by both DOC quantity and quality. However, Ni toxicity varied by less than a factor of 2 among different natural water sources. No clear influence of DOC was found for the mussel *Mytilus galloprovincialis* for DOC in the range 1.2 to 2.7 mg/L or for the diatom *Selenastrum costatum* for DOC in the range 0.2 to 2.7 mg/L (Deforest and Schlekat 2013). Because the effect of DOC on Ni toxicity is limited, marine HC values have not been corrected for bioavailability.

### SSDs and HC values

Relative to many other chemicals, there is now a large data set for Ni toxicity to both temperate and tropical species. Using this data set, the derived marine HC values for different levels of ecosystem protection are shown in Table 4 (Australian and New Zealand Governments 2018). It is the HC5 value that would mostly be applied in slightly to moderately disturbed systems in Australia and New Zealand (Figure 2C and Table 4). Warne et al. (2018) provided guidance for assessing the reliability of HC values derived from SSD methods. This is based on the sample size (number of species for which toxicity data are available), the type of data (chronic, chronic and acute, or converted values), and visual assessment of the fit of the SSD to the toxicity data (i.e., good or poor). The fit of the SSD was good (Figure 2A and C) for both the temperate and combined temperate plus tropical data sets, so, using the classification outlined by Warne et al. (2018), the derived HC values were classified as being of very high reliability (Table 5). The fit of the tropical data set (Figure 2B) was poor, particularly at higher Ni concentrations; and the derived HC values were considered to be of moderate reliability (Table 5).

**Table 5:** Assessment of the reliability of the derived hazardous concentration values

Criterion	Temperate	Tropical	Temperate + tropical
Sample size	24	16	40
Type of toxicity data used in SSD	NOEC, EC10, EC20, EC50/5	NOEC, EC10, LC10, EC50/5	NOEC, EC10, EC20, EC50/5
Assessment of SSD model fit	Good	Poor	Good
Reliability^a^	Very high	Moderate	Very high

^a^ See Warne et al. (2018) for definitions of guideline value reliability.

EC10 = 10% effect concentration; EC50/5 = Chronic 50% effect concentration converted to NOEC value by dividing by 5 (Warne et al. 2018); LC10 = 10% lethal concentration; NOEC = no‐observed‐effect concentration.

It is recommended that the combined data set and HC5 value, 5.8 µg Ni/L (rounded up to 6 µg/L), be used as the basis for a jurisdiction‐specific guideline for Ni for both temperate and tropical marine waters. There was little difference in the overall sensitivity of temperate and tropical species to Ni and in the resultant HC5 values calculated from each SSD. The combined SSD utilizes a larger data set and includes a broader range of species, 40 in total. It is inevitable that the guideline will not be protective of all species. In this instance, 1 sensitive sea urchin and 1 copepod were below the HC5 value, although other sea urchin and copepod species were protected. In addition, this value is above the typically reported background concentrations of Ni in seawater (<5 µg/L; DeForest and Schlekat 2013; Apte et al. 2018).

The HC5 value reported in the present study (6 µg/L) is similar to other guideline values previously reported in Australia and overseas. The current Australian and New Zealand water quality guideline value for Ni (based on temperate marine data) is 70 µg Ni/L, based on 15 species from 5 taxonomic groups. However, because this was insufficiently protective of some species, the default guideline for slightly to moderately disturbed systems was set at 7 µg Ni/L (99% species protection; Australian and New Zealand Environment and Conservation Council and Agriculture and Resource Management Council of Australia and New Zealand 2000). The US Environmental Protection Agency derived a similar chronic Ni guideline for saltwater of 8.2 µg/L. The Ni environmental quality standard (EQS) under the European Union's Water Framework Directive for coastal marine waters is 8.6 µg Ni/L (Nickel Institute 2012). DeForest and Schlekat (2013) undertook further toxicity testing with temperate marine species and provided 2 additional data. In their study, the most sensitive species to Ni was a tropical species of a long‐spined sea urchin (*Diadema antillarum*) from the Caribbean region, which had an EC10 value of 2.9 µg Ni/L. However, this toxicity test was carried out at 20 °C so was not included in our tropical compilation. DeForest and Schlekat (2013) derived a marine Ni HC5 value of 3.9 µg Ni/L (including this tropical sea urchin) and 21 µg Ni/L (when the sea urchin data were excluded because of lack of relevance to European marine waters).

## CONCLUSION

The present study compiled and quality‐checked chronic Ni toxicity data for temperate and tropical marine species. There was little difference in the range of sensitivities of temperate and tropical marine species to Ni and in the resultant HC values that were calculated from the SSDs. As such, and to ensure greater taxonomic coverage, it is recommended that the combined temperate and tropical data set be used to generate a guideline value of 6 µg Ni/L, to ensure the protection of marine species in both temperate and tropical environments.

## Supplemental Data

The Supplemental Data are available on the Wiley Online Library at https://doi.org/10.1002/etc.4880.

## Supporting information

This article includes online‐only Supplemental Data.

Supporting information.

## Data Availability

Data, associated metadata, and calculation tools are available from the corresponding author (fg409@uowmail.edu.au).
