# Supplementary material for: Deriving a Chronic Guideline Value for Nickel in Tropical and Temperate Marine Waters
Source: Environ Toxicol Chem. 2020 Nov 10;39(12):2540–51. doi: 10.1002/etc.4880 (PMC7756218; doi:10.1002/etc.4880)
Supplement: Supplementary file 1 — Supporting information. [file ETC-39-2540-s001.docx]

**Title: Deriving a chronic guideline value for nickel in tropical and temperate marine waters**

**Supplementary information**

**Table S1. Chronic temperate marine nickel toxicity data**

| **Taxonomic Group** | **Species** | | **Life stage** | **Duration** | **Toxicity measure** | **Test medium** | **Temp. (°C)** | **Salinity (‰)** | **pH** | **Concentration (µg/L)** | **Reference** |
| --- | --- | --- | --- | --- | --- | --- | --- | --- | --- | --- | --- |
| Diatom | *Skeletonema costatum* | | _ | 96 h | EC10 (growth) | Seawater | 20 | 28.5 | 8.4 | 142 | Deforest and Schlekat (2013) |
| Diatom | *Skeletonema costatum* | | _ | 96 h | EC10 (growth) | Seawater | 20 | 30.2 | 8.3 | 89 | Deforest and Schlekat (2013) |
| Diatom | *Skeletonema costatum* | | _ | 96 h | EC10 (growth) | Seawater | 20 | 29.4 | 8.2 | 383 | Deforest and Schlekat (2013) |
| Diatom | *Skeletonema costatum* | | _ | 96 h | EC10 (growth) | Seawater | 20 | 29.2 | 8.3 | 190 | Deforest and Schlekat (2013) |
| Diatom | *Skeletonema costatum* | | _ | 96 h | EC10 (growth) | Seawater | 20 | 29.4 | 8.3 | 43.5 | Deforest and Schlekat (2013) |
|  |  | |  |  |  |  | **Geomean of EC10 values** | | | **132** | **Value used in SSD** |
| Green alga | *Dunaliella tertiolecta* | | _ | 96 h | EC10 (growth) | Seawater | 20 | 29.4 | 7.8 | 17890 | Deforest and Schlekat (2013) |
|  |  | |  |  |  |  |  |  |  | **17900** | **Value used in SSD** |
| Red macroalga | *Champia parvula* | | Adult | 10 d | EC10 (reproduction) | Seawater | 23 | 30 | 8 | 144 | Deforest and Schlekat (2013) |
|  |  | |  |  |  |  |  |  |  | **144** | **Value used in SSD** |
| Brown macroalga | *Macrocystis pyrifera* | | Zoospores | 10 d | EC10 (germination) | Seawater | 15 | 34 | 8 | 494 | Golder (2007) |
| Brown macroalga | *Macrocystis pyrifera* | | Zoospores | 10 d | EC10 (reproduction) | Seawater | 15 | 34 | 8 | 96.7 | Golder (2007) |
|  |  | |  |  |  |  |  |  |  | **96.7** | **Value used in SSD** |
| Crustacean | *Mysidopsis intii* | | Neonate | 48 h | NOEC (survival) | Seawater | 20 | 34 | _ | 10 | Hunt et al. (2002) |
| Crustacean | *Mysidopsis intii* | | Neonate | 48 h | EC10 (survival) | Seawater | 20 | 34 | _ | 45.2^a^ | Hunt et al. (2002) |
|  |  | |  |  |  |  |  |  |  | **45.2** | **Value used in SSD** |
| Crustacean | *Mysidopsis bahia* | | Larvae | 36 d | EC50 (reproduction) | Seawater | 23 | 30 | _ | 93 | Gentile et al. (1982) |
| Crustacean | *Mysidopsis bahia* | | Larvae | 36 d | NOEC (reproduction) | Seawater | 23 | 30 | _ | 61 | Gentile et al. (1982) |
| Crustacean | *Mysidopsis bahia* | | Larvae | 20 d | MATC (reproduction) | Seawater | 23 | 30 | _ | 93 | Gentile et al. (1982) |
|  |  | |  |  |  |  |  |  |  | **61** | **Value used in SSD** |
| Crustacean | *Artemia salina* | | Eggs | 48 h | EC50 (hatching rate) | Seawater | 24 | - | - | 4660 | Kissa et al. (1984) |
| Crustacean | *Artemia salina* | | Eggs | 48 h | LOEC (hatching rate) | Seawater | 25 | - | - | 2770 |  |
|  |  | |  |  |  |  |  |  |  | **932** | **EC50/5 value used in SSD** |
| Crustacean | *Litopenaeus vannamei* | | Post-larval | 30 d | EC50 (mortality) | Salinity adjusted seawater | 20 | 25 | 7.0 | 446 | Leonard et al. (2011) |
|  |  | |  |  |  |  |  |  |  | **89** | **EC50/5 value used in SSD** |
| Crustacean | *Excirolana armata* | | Post-larval | 15 d | EC50 (survival) | Seawater | 20 | 25 | 7 | 1350 | Leonard et al. (2011) |
|  |  | |  |  |  |  |  |  |  | **270** | **EC50/5 value used in SSD** |
| Crustacean | *Portunus pelagicus* | | Larve | 42 d | Mean of NOEC and LOEC ((reduced size, moult inhibition) | Seawater | 26 | 33 | - | 32 | Mortimer and Miller (1994) |
|  |  | |  |  |  |  |  |  |  | **32** | **Value used in SSD** |
| Echinoderm | *Diadema antillarum* | | Larvae | 40 h | EC50 (larval development) | Seawater | 20 | 33 | _ | 15 | Bielmyer et al. (2005) |
| Echinoderm | *Diadema antillarum* | | Larvae | 40 h | EC10 (larval development) | Seawater | 20 | 33 | _ | 2.9^a^ | Bielmyer et al. (2005) |
|  |  | |  |  |  |  |  |  |  | **2.9** | **Value used in SSD** |
| Echinoderm | *Paracentrotus lividus* | | Embryo | 72 h | EC50 (fertilisation) | Seawater | 16.3 | 38 | _ | 217 | Pagano et al. (2007) |
| Echinoderm | *Paracentrotus lividus* | | Embryo | 72 h | NOEC (fertilisation) | Seawater | 18 | 35 | _ | 500 | Novelli et al. (2003) |
| Echinoderm | *Paracentrotus lividus* | | Embryo | 72 h | NOEC (larval development) | Seawater | 18 | 35 | _ | 50 | Novelli et al. (2003) |
| Echinoderm | *Paracentrotus lividus* | | Embryo | 72 h | EC50 (larval development) | Seawater | 18 | 35 | _ | 320 | Novelli et al. 2003 |
|  |  | |  |  |  |  |  |  |  | **50** | **Value used in SSD** |
| Echinoderm | *Evichinus chloroticus* | | Embryo | 96 h | EC50 (larval development) | Seawater | 15 | 32 | _ | 14 | Blewett et al. (2017) |
|  |  | |  |  |  |  |  |  |  | **2.8** | **EC50/5 value used in SSD** |
| Echinoderm | *Hemicentrotus pulcherrimus* | | Embryo | 64 h | NOEC (larval development) | Seawater | 16 | 32 | 7.8-8.2 | <10 | Hwang et al. (2012) |
| Echinoderm | *Hemicentrotus pulcherrimus* | | Embryo | 64 h | LOEC (larval development) | Seawater | 16 | 32 | 7.8-8.2 | 25 | Hwang et al. (2012) |
| Echinoderm | *Hemicentrotus pulcherrimus* | | Embryo | 64 h | EC50 (larval development) | Seawater | 16 | 32 | 7.8-8.2 | 34.2 | Hwang et al. (2012) |
|  |  | |  |  |  |  |  |  |  | **6.8** | **EC50/5 value used in SSD** |
| Echinoderm | *Strongeocentrotus purpuratus* | | Embryo | 48 h | EC10 (larval development) | Seawater | 15.6 | 30 | 8.1 | 335 | Deforest and Schlekat (2013) |
|  |  | |  |  |  |  |  |  |  | **335** | **Value used in SSD** |
| Echinoderm | *Dendraster excentricus* | | Embryo | 48 h | EC10 (larval development) | Seawater | 15.4 | 30 | 8.1 | 191 | Deforest and Schlekat (2013) |
|  |  | |  |  |  |  |  |  |  | **191** | **Value used in SSD** |
| Gastropod mollusc | *Haliotis rufescens* | | Embryo | 14 d | NOEC (shell growth) | Seawater | 20 | 34 | _ | 21.5 | Hunt et al 2002 |
| Gastropod mollusc | *Haliotis rufescens* | | Embryo | 14 d | EC10 (shell growth) | Seawater | 20 | 34 | _ | 36.4^a^ | Hunt et al 2002 |
|  |  | |  |  |  |  |  |  |  | **22** | **Value used in SSD** |
| Bivalve mollusc | *Crassostrea gigas* | | Embryo | 96 h | EC10 (reproduction) | Seawater | 20.7 | 30 | 7.4 | 431 | Deforest and Schlekat (2013) |
|  |  | |  |  |  |  |  |  |  | **431** | **Value used in SSD** |
| Bivalve mollusc | *Mytilus edulis* | | Embryo | 96 h | EC50 (development) | Seawater | 17 | 34 | 8.1 | 891 | Martin et al. (1981) |
|  |  | |  |  |  |  |  |  |  | **178** | **EC50/5 value used in SSD** |
| Bivalve mollusc | *Mytilus trossolis* | | Embryo | 48 h | EC20 (survival) | Seawater | 22-25 | 34 | 8 | 88 | Nadella et al. (2009) |
|  |  | |  |  |  |  |  |  |  | **88** | **Value used in SSD** |
| Bivalve mollusc | *Mytilus galloprovincialis* | | Embryo | 48 h | EC10 (survival) | Seawater | 15.8 | 30 | 8.1 | 259 | Deforest and Schlekat (2013) |
| Bivalve mollusc | *Mytilus galloprovincialis* | | Embryo | 48 h | EC10 (survival) | Seawater | 16.1 | 30 | 7.9 | 228 | Deforest and Schlekat (2013) |
| Bivalve mollusc | *Mytilus galloprovincialis* | | Embryo | 48 h | EC10 (survival) | Seawater | 16 | 30 | 8.1 | 256 | Deforest and Schlekat (2013) |
| Bivalve mollusc | *Mytilus galloprovincialis* | | Embryo | 48 h | EC10 (survival) | Seawater | 16.1 | 30 | 8.1 | 350 | Deforest and Schlekat (2013) |
|  |  | |  |  |  |  | **Geomean of EC10 values** | | | **270** | **Value used in SSD** |
| Polychaete | *Neanthes arenaceodentata* | | Adult | 90 d | EC10 (reproduction) | Seawater | 20 | 29.5 | 7.9 | 22.5 | Deforest and Schlekat (2013) |
|  |  | |  |  |  |  |  |  |  | **22** | **Value used in SSD** |
| Fish | *Atherinops affinis* | | Embryo | 40 d | NOEC (larval survival) | Seawater | 20 | 34 | _ | 3240 | Hunt et al. (2002) |
| Fish | *Atherinops affinis* | | Embryo | 40 d | EC10 (larval survival) | Seawater | 20 | 34 | _ | 3600 | Hunt et al. (2002) |
|  |  | |  |  |  |  |  |  |  | **3240** | **Value used in SSD** |
| Fish | | *Cyprinidon variegatus* | Juvenile | 28 d | EC10 (growth) | Seawater | 25 | 28-30 | 8.1 | 20300 | Golder (2007) |
|  |  | |  |  |  |  |  |  |  | **20300** | **Value used in SSD** |

^a^ EC10 from DeForest and Schlekat (2013) using data supplied by authors

**Table S2. Chronic nickel toxicity data for tropical marine species**

| **Taxonomic Group** | **Species** | **Life stage** | **Duration** | **Toxicity measure** | **Test medium** | **Temp. (°C)** | **Salinity (‰)** | **pH** | **Concentration (µg/L)** | **Reference** |
| --- | --- | --- | --- | --- | --- | --- | --- | --- | --- | --- |
| Cyanobacteria | *Cyanobium* sp. | 6 x10^3^ cells/mL | 72 h | EC10  (growth rate) | Seawater with media | 25 | 33 | 8 | 3700 | Alquezar and Anastasi (2013) |
| Cyanobacteria | *Cyanobium* sp. | 7 x10^3^ cells/mL | 72 h | EC50  (growth rate) | Seawater with media | 25 | 33 | 8 | 22500 | Alquezar and Anastasi (2013) |
|  |  |  |  |  |  |  |  |  | **3700** | **Value used in SSD** |
| Diatom | *Ceratoneis closterium (G2)* | 5-6 d old, 1-3 x10^3^ cells/mL | 72 h | NOEC  (growth rate) | Seawater | 27 | 35 | 8.1 | 3970 | Gissi (2018) |
| Diatom | *Ceratoneis closterium (G2)* | 5-6 d old, 1-3 x10^3^ cells/mL | 72 h | EC10  (growth rate) | Seawater | 27 | 35 | 8.1 | 3250 | Gissi (2018) |
| Diatom | *Ceratoneis closterium (F2)* | 5-6 d old, 1-3 x10^3^ cells/mL | 72 h | NOEC  (growth rate) | Seawater | 27 | 35 | 8.1 | 1610 | Gissi (2018) |
| Diatom | *Ceratoneis closterium (F2)* | 5-6 d old, 1-3 x10^3^ cells/mL | 72 h | EC10  (growth rate) | Seawater | 27 | 35 | 8.1 | 2539 | Gissi (2018) |
|  |  |  |  |  |  | **Geomean of EC10 values** | | | **2870** | **Value used in SSD** |
| Brown-golden alga | *Tisochrysis lutea* | 5-6 d old, 1-3 x10^3^ cells/mL | 72 h | NOEC  (growth rate) | Seawater | 27 | 35 | 8.1 | 250 | Gissi (2018) |
| Brown-golden alga | *Tisochrysis lutea* | 5-6 d old, 1-3 x10^3 cells/mL | 72 h | EC10  (growth rate) | Seawater | 27 | 35 | 8.1 | 330 | Gissi (2018) |
|  |  |  |  |  |  |  |  |  | **330** | **Value used in SSD** |
| Dinoflagellate | *Symbiodinium* sp Freud. Clade C. | 6-7 d old, 1-3 x10^3^ cells/mL | 72 h | NOEC  (growth rate) | Seawater | 27 | 35 | 8.1 | 310 | Gissi (2018) |
|  |  |  |  |  |  |  |  |  | **310** | **Value used in SSD** |
| Crustacean | *Amphibalanus amphitrite* | Nauplii  (<2 h old) | 96 h | EC20 (metamorphosis) | Seawater | 29 | 35 | 8.3 | 97 | Gissi et al. (2018) |
| Crustacean | *Amphibalanus amphitrite* | Nauplii  (<2 h old) | 96 h | EC10 (metamorphosis) | Seawater | 29 | 35 | 8.3 | 67 | Gissi et al. (2018) |
|  |  |  |  |  |  |  |  |  | **67** | **Value used in SSD** |
| Copepod | *Acartia pacifica* | Adult females | 10 d | LOEC (egg production) | Seawater | 25 | 25 | 8.1 | 100 | Mohammed et al. (2010) |
|  |  |  |  |  |  |  |  |  | **40** | **LOEC/2.5 value used in SSD** |
| Copepod | *Acartia sinjiensis* | Egg | 80 h | EC20 (development) | Seawater | 30 | 35 | 8.1 | 6.6 | Gissi et al. (2018) |
| Copepod | *Acartia sinjiensis* | Egg | 80 h | EC10 (development) | Seawater | 30 | 35 | 8.1 | 5.5 | Gissi et al. (2018) |
|  |  |  |  |  |  |  |  |  | **5.5** | **Value used in SSD** |
| Copepod | *Tigriopus japonicus* | Nauplii  (<24 h old) | 20-30 d | LC10  (mortality) | Artificial seawater | 27 | 33 | 8.2 | 484 | Wang et al. (2021) |
| Copepod | *Tigriopus japonicus* | Nauplii  (<24 h old) | 20-30 d | NOEC  (mortality) | Artificial seawater | 27 | 33 | 8.2 | 99.8 | Wang et al. (2021) |
| Copepod | *Tigriopus japonicus* | Nauplii  (<24 h old) | 20-30 d | LOEC  (mortality) | Artificial seawater | 27 | 33 | 8.2 | 200 | Wang et al. (2021) |
| Copepod | *Tigriopus japonicus* | Maturation stage | 20-30 d | LOEC  (mortality) | Artificial seawater | 27 | 33 | 8.2 | 99.8 | Wang et al. (2021) |
| Copepod | *Tigriopus japonicus* | Maturation stage | 20-30 d | NOEC  (mortality) | Artificial seawater | 27 | 33 | 8.2 | 50.3 | Wang et al. (2021) |
| Copepod | *Tigriopus japonicus* | Maturation stage | 20-30 d | LC10  (mortality) | Artificial seawater | 27 | 33 | 8.2 | 43.9 | Wang et al. (2021) |
| Copepod | *Tigriopus japonicus* | Nauplii (<24-h old) | 20-30 d | EC10 (Intrinsic rate of increase^a^) | Artificial seawater | 27 | 33 | 8.2 | 29.1 | Wang et al. (2021) |
| Copepod | *Tigriopus japonicus* |  | 20-30 d | EC20 (Intrinsic rate of increase) | Artificial seawater | 27 | 33 | 8.2 | 66.5 | Wang et al. (2021) |
| Copepod | *Tigriopus japonicus* |  | 20-30 d | EC50 (Intrinsic rate of increase) | Artificial seawater | 27 | 33 | 8.2 | 277 | Wang et al. (2021) |
| Copepod | *Tigriopus japonicus* |  | 20-30 d | NOEC (Intrinsic rate of increase) | Artificial seawater | 27 | 33 | 8.2 | 50.3 | Wang et al. (2021) |
| Copepod | *Tigriopus japonicus* |  | 20-30 d | LOEC (Intrinsic rate of increase) | Artificial seawater | 27 | 33 | 8.2 | 99.8 | Wang et al. (2021) |
|  |  |  |  |  |  |  |  |  | **29.1** | **Value used in SSD** |
| Gastropod mollusc | *Nassarius dorsatus* | Larvae  (2 d old) | 96 h | EC20  (growth rate) | Seawater | 28 | 35 | 8.2 | 143 | Gissi et al. (2018) |
| Gastropod mollusc | *Nassarius dorsatus* | Larvae  (2 d old) | 96 h | EC10  (growth rate) | Seawater | 28 | 35 | 8.2 | 64 | Gissi et al. (2018) |
|  |  |  |  |  |  |  |  |  | **64** | **Value used in SSD** |
| Gastropod mollusc | *Monodonta labio* | juvenile  (<10 d old) | 30 d | LC10  (mortality) | Artificial seawater | 27 | 33 | 8.2 | 57 | Wang et al. (2021) |
| Gastropod mollusc | *Monodonta labio* | juvenile  (<10 d old) | 30 d | EC10  (growth rate) | Artificial seawater | 27 | 33 | 8.2 | 33.6 | Wang et al. (2021) |
| Gastropod mollusc | *Monodonta labio* | juvenile  (<10 d old) | 30 d | EC20  (growth rate) | Artificial seawater | 27 | 33 | 8.2 | 58.5 | Wang et al. (2021) |
| Gastropod mollusc | *Monodonta labio* | juvenile  (<10 d old) | 30 d | EC50  (growth rate) | Artificial seawater | 27 | 33 | 8.2 | 151 | Wang et al. (2021) |
| Gastropod mollusc | *Monodonta labio* | juvenile  (<10 d old) | 30 d | NOEC  (growth rate) | Artificial seawater | 27 | 33 | 8.2 | 21.7 | Wang et al. (2021) |
| Gastropod mollusc | *Monodonta labio* | juvenile  (<10 d old) | 30 d | LOEC  (growth rate) | Artificial seawater | 27 | 33 | 8.2 | 53.9 | Wang et al. (2021) |
| Gastropod mollusc | *Monodonta labio* | juvenile  (<10 d old) | 30 d | EC10 (shell length increment) | Artificial seawater | 27 | 33 | 8.2 | 93.5 | Wang et al. (2021) |
| Gastropod mollusc | *Monodonta labio* | juvenile  (<10 d old) | 30 d | EC20 (shell length increment) | Artificial seawater | 27 | 33 | 8.2 | 145 | Wang et al. (2021) |
| Gastropod mollusc | *Monodonta labio* | juvenile  (<10 d old) | 30 d | EC50 (Shell length increment) | Artificial seawater | 27 | 33 | 8.2 | 308 | Wang et al. (2021) |
| Gastropod mollusc | *Monodonta labio* | juvenile  (<10 d old) | 30 d | NOEC (shell length increment) | Artificial seawater | 27 | 33 | 8.2 | 53.9 | Wang et al. (2021) |
| Gastropod mollusc | *Monodonta labio* | juvenile (<10 d old) | 30 d | LOEC (shell length increment) | Artificial seawater | 27 | 33 | 8.2 | 107 | Wang et al. (2021) |
|  |  |  |  |  |  |  |  |  | **33.6** | **Value used in SSD** |
| Coral | *Acropora digitifera* | Gametes | 5 h | NOEC (fertilisation) | Seawater | 25 | 34 | 8.1 | 940 | Gissi et al. (2017) |
| Coral | *Acropora digitifera* | Gametes | 5 h | EC10 (fertilisation) | Seawater | 25 | 34 | 8.1 | 2000 | Gissi et al. (2017) |
| Coral | *Acropora digitifera* | Gametes | 5 h | EC5  (fertilisation) | Seawater | 25 | 34 | 8.1 | 1680 | Gissi et al. (2017) |
|  |  |  |  |  |  |  |  |  | **2000** | **Value used in SSD** |
| Coral | *Platygyra daedalea* | Gametes | 5 h | NOEC (fertilisation) | Seawater | 25 | 34 | 8.1 | 920 | Gissi et al. (2017) |
| Coral | *Platygyra daedalea* | Gametes | 5 h | EC50 (fertilisation) | Seawater | NR | NR | NR | 1420 | Reichelt-Brushett and Hudspith (2016) |
|  |  |  |  |  |  |  |  |  | **920** | **Value used in SSD** |
| Sea anemone | *Exaiptasia pulchella* | Lacerate tentacle | 14 d | EC10 (development) | Seawater | 25 | 34 | 8.2 | 260 | Howe et al. (2014) |
| Sea anemone | *Exaiptasia pulchella* | Adults | 28 d | EC10 (reproduction - total number of offspring) | Seawater | 25 | NR | 8.2 | 260 | Howe et al. (2014) |
| Sea anemone | *Exaiptasia pulchella* | Adult | 28 d | EC50 (reproduction - total number of offspring) | Seawater | 25 | NR | 8.2 | 400 | Howe et al. (2014) |
| Sea anemone | *Exaiptasia pulchella* | Adult | 28 d | LOEC (reproduction - total number of offspring) | Seawater | 25 | NR | 8.2 | 510 | Howe et al. (2014) |
| Sea anemone | *Exaiptasia pulchella* | Adult | 28 d | EC10 (reproduction - total number of juveniles) | Seawater | 25 | NR | 8.2 | 65 | Howe et al. (2014) |
| Sea anemone | *Exaiptasia pulchella* | Adult | 28 d | EC50 (reproduction - total number of juveniles) | Seawater | 25 | NR | 8.2 | 370 | Howe et al. (2014) |
| Sea anemone | *Exaiptasia pulchella* | Adult | 28 d | LOEC (reproduction - total number of juveniles) | Seawater | 25 | NR | 8.2 | 510 | Howe et al. (2014) |
|  |  |  |  |  |  |  |  |  | **65** | **Value used in SSD** |
| Echinoderm | *Diadema savignyi* | Gametes | 48 h | EC50  (fertilisation and development) | Seawater | 25 | 34 | 8.1 | 117 | Rosen et al. (2015) |
| Echinoderm | *Diadema savignyi* | Gametes | 48 h | LOEC  (fertilisation and development) | Seawater | 25 | 34 | 8.1 | 36.5 | Rosen et al. (2015) |
| Echinoderm | *Diadema savignyi* | Gametes | 48 h | NOEC (fertilisation and development) | Seawater | 25 | 34 | 8.1 | 23.5 | Rosen et al. (2015) |
| Echinoderm | *Diadema savignyi* | Gametes | 48 h | EC50  (fertilisation and development) | Seawater | 25 | 34 | 8.1 | 71.6 | Rosen et al. (2015) |
| Echinoderm | *Diadema savignyi* | Gametes | 48 h | LOEC  (fertilisation and development) | Seawater | 25 | 34 | 8.1 | 36.5 | Rosen et al. (2015) |
| Echinoderm | *Diadema savignyi* | Gametes | 48 h | NOEC (fertilisation and development) | Seawater | 25 | 34 | 8.1 | 22.5 | Rosen et al. (2015) |
|  |  |  |  |  |  | **Geomean of NOEC values** | | | **23** | **Value used in SSD** |
| Polychaete | *Hydroides elegans* | Gametes | 1 h | EC50 (sperm viability/fertilisation) | Seawater | 28 | 34 | 8.1 | 773 | Gopalakrishnan et al. (2008) |
| Polychaete | *Hydroides elegans* | Gametes | 1 h | EC50 (egg viability/fertilisation) | Seawater | 28 | 34 | 8.1 | 1178 | Gopalakrishnan et al. (2008) |
| Polychaete | *Hydroides elegans* | Gametes | 2 h | EC50 (embryo development) | Seawater | 28 | 34 | 8.1 | 2263 | Gopalakrishnan et al. (2008) |
| Polychaete | *Hydroides elegans* | Adults | 20 h | EC50 (larval release) | Seawater | 28 | 34 | 8.1 | 410 | Gopalakrishnan et al. (2008) |
| Polychaete | *Hydroides elegans* | Larvae (trochophore stage) | 96 h | EC50 (larval settlement) | Seawater | 28 | 34 | 8.1 | 160 | Gopalakrishnan et al. (2008) |
|  |  |  |  |  |  |  |  |  | **32** | **EC50/5 value used in SSD** |
| Fish | *Oryzias melastigma* | juvenile (1-month post hatching) | 21 d | LC10  (mortality) | Artificial seawater | 27 | 30 | 8.94-8.98 | 1660 | Wang et al. (2021) |
| Fish | *Oryzias melastigma* | juvenile (1-month post hatching) | 21 d | LC20  (mortality) | Artificial seawater | 27 | 30 | 8.94-8.99 | 2310 | Wang et al. (2021) |
| Fish | *Oryzias melastigma* | juvenile (1-month post hatching) | 21 d | LC50  (mortality) | Artificial seawater | 27 | 30 | 8.94-8.100 | 4060 | Wang et al. (2021) |
|  |  |  |  |  |  |  |  |  | **1660** | **Value used in SSD** |

^a^Intrinsic rate of increase =Population growth = number of births-number of deaths

**Table S3.** Comparison of HCx values calculated from the Burr Type III and Log-normal distributions

| Protection Level (HC) (%) | Toxicity value, µg Ni/L (95% confidence limits) | | | | | |
| --- | --- | --- | --- | --- | --- | --- |
|  | Temperate | | Tropical | | Temperate + tropical | |
|  | Burr Type III | Log-normal | Burr Type III | Log-normal | Burr Type III | Log-normal |
| 1% | 1.2 (0.14-7.4) | 1.2 (0.44-3.5) | 4.6 (0.03-15) | 0.18 (0.02-1.4) | 1.8 (0.43-7.2) | 0.45 (0.27-0.75) |
| 5% | 4.4 (1.8-17) | 6.2 (2.9-13) | 9.6 (1.7-26) | 2.1 (0.5-8.3) | 5.8 (2.8-15) | 3.35 (2.3-4.8) |
| 10% | 8.7 (3.9-27) | 13 (6.7-24) | 15 (7.1-41) | 6.2 (2-19) | 11 (5.6-25) | 8.3 (6.2-11) |
| 20% | 20 (8.1-53) | 28 (16-49) | 28 (13-89) | 20 (8.8-48) | 23 (12-47) | 23 (18-29) |

**Table S4.** Comparison of log-normal species sensitivity distributions (SSDs: 𝑦=*a*𝑥+*b*, slope *a* and *y*-intercept *b*) using analysis of covariance (ANCOVA, level of significance α = 0.05). Both Anderson-Darling test (critical value *A****^2^* =** 1.933; under 10% significance level) test and Shapiro-Francia test (critical *W'* = 1.645 under 5% significance level) were used for normality check (A) of each dataset. Results from ANCOVA analysis shown in (B).

1. **Normality check**

| SSDs | Anderson-Darling test | Shapiro-Francia test | |
| --- | --- | --- | --- |
|  | Statistic *A****^2^*** | Statistic *W'* | *p* value |
| Temperate | 1.332 | 0.517 | 0.302 |
| Tropical | 0.462 | -0.822 | 0.794 |
| Combined | 0.52 | -0.641 | 0.739 |

1. **ANCOVA analysis**

| SSD comparisons | y = *ax* + *b* | | |
| --- | --- | --- | --- |
|  | Statistic | a | b |
| Temperate vs Tropical | F*_1,35_* = 209 | p<0.05 | NA |
| Temperate vs Combined | F*_1,56_* = 509 | p<0.05 | NA |
| Tropical vs Combined | F*_1,50_* = 611 | p<0.05 | NA |

**Figure S1** Cumulative distribution of chronic nickel toxicity data for tropical, temperate and combined datasets
